# Hypoxia promotes breast cancer cell invasion through HIF-1α-mediated up-regulation of the invadopodial actin bundling protein CSRP2

**DOI:** 10.1038/s41598-018-28637-x

**Published:** 2018-07-05

**Authors:** Céline Hoffmann, Xianqing Mao, Joshua Brown-Clay, Flora Moreau, Antoun Al Absi, Hannah Wurzer, Barbara Sousa, Fernando Schmitt, Guy Berchem, Bassam Janji, Clément Thomas

**Affiliations:** 1 Laboratory of Experimental Cancer Research, 84 Val Fleuri, L-1526 Luxembourg, Luxembourg; 2 Faculaty of Science, Technology and Communication, University of Luxembourg, 2 avenue de l’Université, L-4365 Esch-sur-Alzette, Luxembourg; 3 IPATIMUP- Institute of Molecular Pathology and Immunology of the University of Porto, Medical Faculty of Porto University, Rua Julio Amaral de Carvalho 45, 4200-135 Porto, Portugal

## Abstract

Hypoxia is a common feature of solid tumours that promotes invasion and metastatic dissemination. Invadopodia are actin-rich membrane protrusions that direct extracellular matrix proteolysis and facilitate tumour cell invasion. Here, we show that CSRP2, an invadopodial actin bundling protein, is upregulated by hypoxia in various breast cancer cell lines, as well as in pre-clinical and clinical breast tumour specimens. We functionally characterized two hypoxia responsive elements within the proximal promoter of CSRP2 gene which are targeted by hypoxia-inducible factor-1 (HIF-1) and required for promoter transactivation in response to hypoxia. Remarkably, CSRP2 knockdown significantly inhibits hypoxia-stimulated invadopodium formation, ECM degradation and invasion in MDA-MB-231 cells, while CSRP2 forced expression was sufficient to enhance the invasive capacity of HIF-1α-depleted cells under hypoxia. In MCF-7 cells, CSRP2 upregulation was required for hypoxia-induced formation of invadopodium precursors that were unable to promote ECM degradation. Collectively, our data support that CSRP2 is a novel and direct cytoskeletal target of HIF-1 which facilitates hypoxia-induced breast cancer cell invasion by promoting invadopodia formation.

## Introduction

Metastasis, i.e. the spread of tumour cells from the primary tumour and subsequent colonization of distant organs, is the most life-threatening aspect of cancer^1^. The hypoxic tumour microenvironment is a potent driver of tumour aggressiveness and metastasis, and is highly associated with poor clinical outcomes in various cancers^2–4^. A fundamental process underlying the pro-metastatic effect of hypoxia is the stimulation of tumour cell invasive capabilities. At the subcellular level, hypoxia has recently been reported to promote the formation of actin-rich membrane protrusions, termed invadopodia^5^. Invadopodia facilitate tumour cell invasion through dense extracellular matrix (ECM) by recruiting transmembrane and secreted metalloproteinases (MMPs) that catalyze ECM component degradation, and creating pores through which mesenchymal tumour cells can migrate^6,7^. Both *ex vivo* and *in vivo* studies have provided direct evidence of the critical roles of invadopodia during key steps of the metastatic cascade, such as basement membrane breaching, intravasation and extravasation^8–12^. In addition, it has been suggested that invadopodia may contribute to other important aspects of disease progression, such as tumour growth and angiogenesis^13,14^, further increasing interest in their potential as therapeutic targets.

Invadopodium biogenesis largely relies on cytoskeletal rearrangements orchestrated by a combination of lamellipodial and filopodial actin machineries^15–18^. A critical step of invadopodium initiation is the assembly of an actin core by the ARP2/3 complex and its associated regulators, such as N-WASP and cortactin. Invadopodium elongation is promoted by the expansion of the actin core in both branched networks and unbranched bundles. At the tip of invadopodia, actin bundles presumably potentiate the protrusive force generated by actin polymerization, whereas the dendritic actin network progressively expands to fill and stabilize upstream regions^16,18^. The actin cytoskeleton proteins and upstream signalling pathways involved in invadopodium biogenesis have been characterized to a great extent^7^. However, our understanding of how important components of the tumour microenvironment, such as hypoxia, shape the invasive behavior of tumour cells remains fragmented^5,7^.

Cysteine-rich protein 2 (CSRP2) is a short (21 kDa) two LIM domain-containing protein, which is upregulated in invasive breast cancer cells, and localizes along the protrusive actin core of invadopodium^19^. Similar to its relatives CSRP1 and CSRP3/muscle LIM protein^20,21^, CSRP2 crosslinks actin filaments in stable bundles, suggesting that it contributes to the assembly and/or maintenance of the invadopodium actin backbone^19^. Accordingly, CSRP2 knockdown significantly inhibits invadopodium formation in aggressive breast cancer cells, as well as MMP secretion and 3D matrix invasion. It also strongly reduces tumour cell dissemination in two mouse models of breast cancer metastasis. The clinical relevance of these findings to human breast cancer disease is supported by microarray data identifying *CSRP2* in a cluster of 14 upregulated genes characteristic of the highly aggressive basal-like breast carcinoma subtype^22^. In addition, among basal-like tumour patients, those with high CSRP2 expression exhibit an increased risk for developing metastasis. In the present study, we show that hypoxia upregulates CSRP2 in different breast cancer cell lines, and that such upregulation results from HIF-1-mediated transactivation of the CSRP2 promoter. We provide evidence that CSRP2 depletion strongly reduces the ability of hypoxia to enhance invadopodia formation, ECM degradation and invasion in highly invasive breast carcinoma cell lines, such as MDA-MB-231 and mouse 4T1. In weakly invasive, epithelial-like, MCF-7 cells, hypoxia-induced CSRP2 expression was required for the formation of invadopodium precursors, which were unable to promote ECM digestion due to the lack of MT1-MMP expression. Finally, we found that CSRP2 up-regulation correlates with hypoxic regions in both pre-clinical and clinical breast tumour specimens, and is associated with poor prognosis in breast cancer patients. Overall, our data point to an important role for CSRP2 in facilitating the pro-invasive and -metastatic effects of hypoxia in breast cancer.

## Results

### Hypoxia promotes HIF-1 dependent CSRP2 up-regulation in breast cancer cells

**Figure 1:**
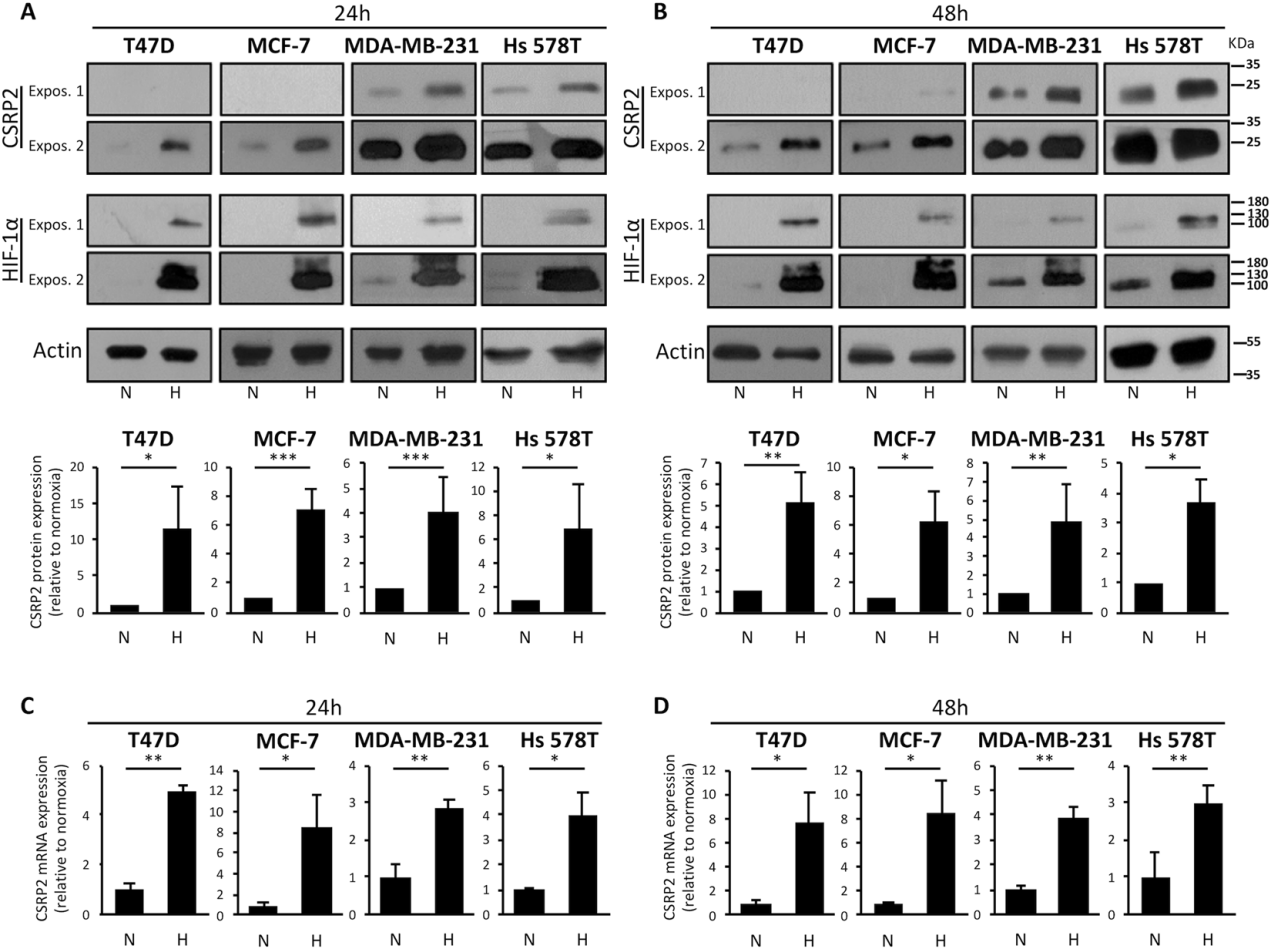
Hypoxia upregulates CSRP2 protein and transcript levels in human breast cancer cells. (**A**,**B**) Western blot analysis of CSRP2 and HIF-1α protein levels in epithelial (T47D, MCF-7) and mesenchymal (MDA-MB-231, HS-578T) human breast cancer cells cultured for 24 h (**A**) or 48 h (**B**) in normoxic (N) or hypoxic (H) conditions. Short and long exposures for CSRP2 and HIF-1α blots are shown to better appreciate the differences between the cell lines (“Expos. 1” and “2”, respectively). After quantification, protein levels were normalized to β-actin levels and CSRP2 protein levels in hypoxia were expressed as fold of normoxic control (set to 1). The lower charts indicate the mean ± s.e. calculated from at least 4 independent experiments. (**C**,**D**) qRT-PCR analysis of CSRP2 transcript levels in the same four cell lines after 24 h (**C**) or 48 h (**D**) incubation in normoxic (N) or hypoxic (H) conditions. Results are expressed as CSRP2 transcript levels in hypoxia relative to normoxic control (set to 1). Shown are the mean ± s.e. calculated from 3 (**C**) and 4 (**D**) independent experiments. *p < 0.05; **p < 0.01; ***p < 0.001.

The hypoxic tumour microenvironment is a critical promoter of breast cancer progression and metastasis^3,23^. We assessed the effects of hypoxia on the expression of the pro-invasive and -metastatic invadopodial protein CSRP2 in four breast cancer cell lines, including luminal/epithelial-like MCF-7 and T47D (ER^+^, PR^+^), and mesenchymal-like MDA-MB-231 and Hs578T (ER^−^, PR^−^, HER2^−^, claudin-low). In agreement with our previous report^19^, CSRP2 was absent or only weakly expressed in epithelial-like cells under normoxia, whereas it was expressed at significant levels in mesenchymal-like cells (Fig. 1A and B). Exposing cells to hypoxia (0.1% p0_2_) for 24 hours induced a significant up-regulation of CSRP2 in all four cell lines (Fig. 1A). Indeed, CSRP2 protein levels increased by about ten times in epithelial-like cells and by about five times in mesenchymal-like cells, as compared to the respective normoxic controls. At 48 hours of hypoxia, high CSRP2 protein expression was maintained in all cell lines (Fig. 1B). To extend these data, *CSRP2* transcript levels were analysed in normoxic and hypoxic conditions using real-time qRT-PCR. In the four breast cancer cell lines, hypoxia induced a significant and sustained elevation of *CSRP2* transcripts (Fig. 1C and D), suggesting that CSRP2 levels were regulated at the transcriptional level.

**Figure 2:**
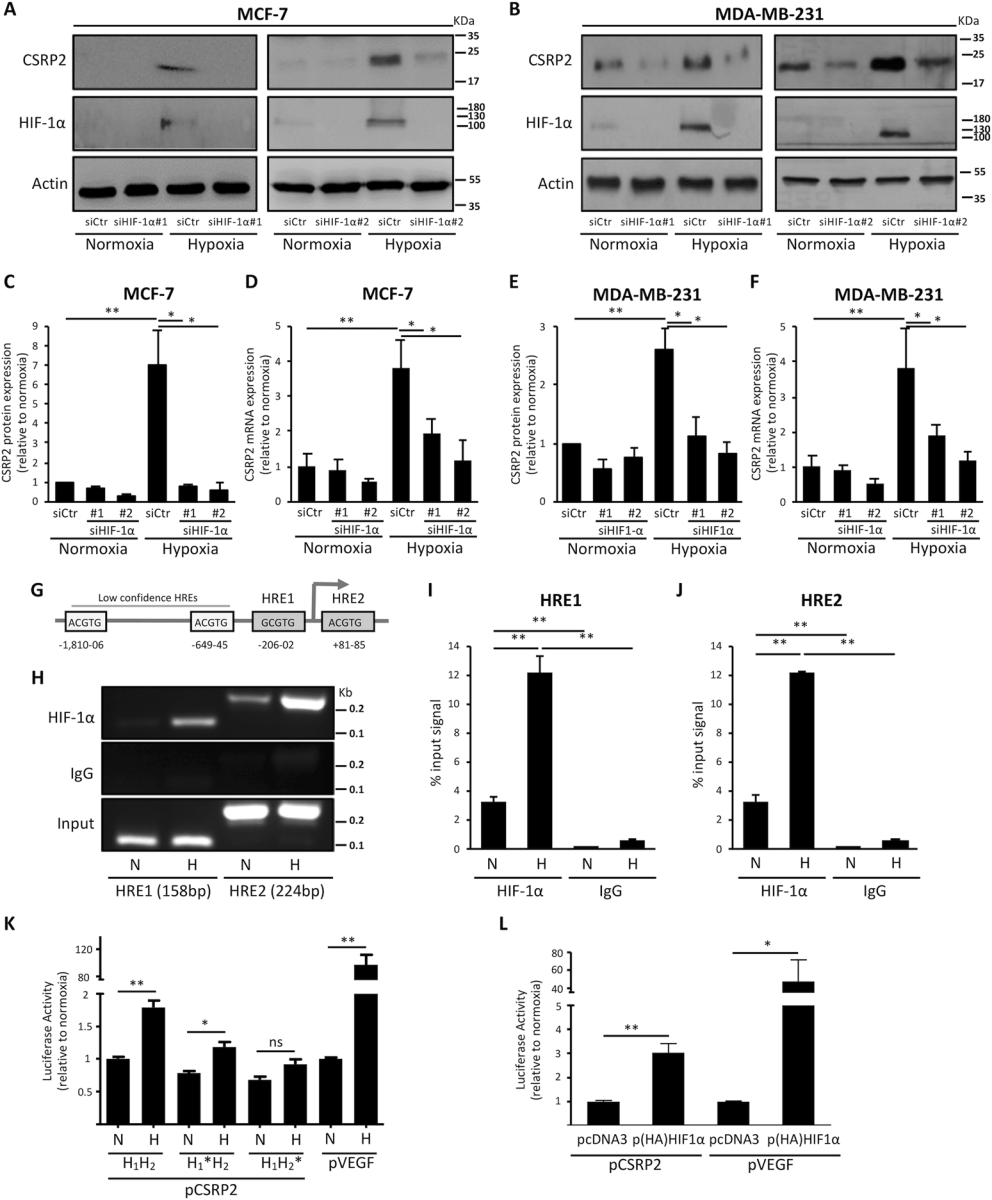
HIF-1α regulates CSRP2 promoter activity. (**A**,**B**) Western blot analysis of CSRP2 and HIF-1α protein levels in MCF-7 (**A**) and MDA-MB-231 (**B**) breast cancer cells incubated in normoxia or hypoxia and transfected with control siRNAs (siCtr) or two different HIF-1α targeting siRNAs (siHIF-1α#1 and #2). (**C**,**E**) After quantification, protein levels were normalized to β-actin levels and CSRP2 and HIF-1α protein levels were expressed relative to siCtr-transfected normoxic control (set to 1). Shown are the mean ± s.e. calculated from four (**C**) and five (**E**) independent experiments. (**D**,**F**) qRT-PCR analysis of CSRP2 transcript levels in the same cell lines and culture conditions. Results are expressed as CSRP2 transcript levels in hypoxia relative to siCtr-transfected normoxic control (set to 1). Shown are the mean ± s.e. calculated from three (**D**) and four (**F**) independent experiments. (**G**) A schematic representation of the putative HREs identified in the proximal promoter of CSRP2. The position (relative to the transcriptional start site; TSS) of low and high confidence HREs is indicated. (**H**) ChiP analysis for HIF-1α recruitment to CSRP2 promoter regions containing HRE1 and HRE2 in MCF-7 cells cultured in normoxic (N) or hypoxic (H) conditions. (**I**,**J**) Quantification of ChiP data for HRE1 (G) and HRE2 (H) from three independent experiments. Data are expressed as percentage of PCR product signal obtained for chromatin fragments immunoprecipitated with HIF-1α antibodies or IgG (non-specific binding control) relative to the corresponding input signal. (**K**) Luciferase reporter assays to evaluate the normoxic and hypoxic activity of a wild type CSRP2 promoter with intact HRE1 and HRE2 (H1H2) and two variants with point mutations in HRE1 (H1*H2) or HRE2 (H1H2*) in MCF-7 cells. VEGF promoter was used as a positive control. Data are expressed as mean of luciferase signal ± s.e. in hypoxia relative to normoxic control (set to 1; n = 3 independent experiments). (**L**) Luciferase assays for the wild type CSRP2 promoter in MCF-7 cells transfected with a control empty plasmid (pcDNA3) or HIF-1α expressing plasmid (p(HA) HIF-1α). Data are expressed as mean of luciferase signal ± s.e. in hypoxia relative to normoxic control (set to 1; independent experiments). *p < 0.05; **p < 0.01.

Tumour cells respond to hypoxic stress by an activation of HIFs, which drive the transcription of target genes through binding to cis-regulatory elements, termed hypoxia responsive elements (HREs)^24^. Accumulating evidence indicates that HIF-1 functions as a master regulator of the hypoxic response in breast cancer^2,23^. To evaluate the role of HIF-1 in hypoxia-induced CSRP2 expression, its oxygen-regulated subunit (HIF-1α) was depleted in MCF-7 cells prior exposure to hypoxia. HIF-1α knockdown significantly inhibited hypoxia-induced CSRP2 up-regulation at both protein and mRNA levels (Fig. 2A,C and D). Similar results were obtained by knocking down HIF-1α in MDA-MB-231 cells (Fig. 2B,E and F), supporting that the gene encoding CSRP2 is a novel, and direct downstream target of HIF-1.

### HIF-1α drives CSRP2 transcription by direct binding to HRE motifs

The consensus core HRE sequence 5′-[A/G]CGT-3′ was initially characterized based on the comparison of various known HIF target genes^25^. Later on, flanking nucleotides were demonstrated to contribute additional information to the likelihood of HIF-1 transactivation^26^. *In silico* analysis using the weight matrix-based program *Alggen PROMO*^27^, identified two high-confidence HREs (HRE1 and HRE2) in the proximal promoter region of *CSRP2*, while manual scanning found two additional lower confidence core HRE sequences further upstream (Fig. 2G).

To validate HIF-1α binding to these two putative HREs, we conducted ChIP analyses, using HIF-1α specific antibody (Fig. 2H). Results revealed that HIF-1α significantly binds to both HREs, even under normoxic conditions. Additionally, there was a dramatic and significant increase (>12 fold) in HIF-1α occupancy at these sites under hypoxia (Fig. 2H–J).

To study the transactivation of *CSRP2* by HIF-1α, the nucleotides −1,920 to +103 relative to the transcription start site were cloned into a luciferase reporter vector. MCF-7 cells were chosen to conduct the reporter assays as they express low levels of CSRP2 and HIF-1α under normoxic conditions (Fig. 2A). As shown in Fig. 2K, hypoxia induced a significant increase in luciferase activity as compared to normoxia. A *VEGFA* promoter luciferase reporter was used as a positive control of hypoxia-induced gene transcription. Confirming the ChIP results, mutation of HRE1 decreased the hypoxic induction of transcriptional activity at the *CSRP2* promoter, while mutation of HRE2 reduced this induction to an insignificant amount (p = 0.1237). These results suggest that the HRE2 sequence is the major motif responsible for hypoxia-dependent induction of *CSRP2* expression, while HRE1 may also contribute to a lesser extent.

To further confirm that the *CSRP2* promoter is transactivated by HIF-1α, constitutively stable HIF-1α was co-transfected into MCF-7 cells along with the luciferase reporter plasmids. Again, a *VEGFA* promoter luciferase reporter construct was used as a positive control of HIF-1α transcriptional activity. As shown in Fig. 2L, HIF-1α induced a significant transactivation of the *CSRP2* promoter, supporting its role in hypoxia-promoted *CSRP2* expression.

Taken together, ChIP and luciferase reporter experiments show that hypoxia induces binding of the HIF-1α transcription factor to the *CSRP2* promoter, primarily at the HRE2 site, and subsequent induction of *CSRP2* expression.

### CSRP2 mediates hypoxia-stimulated invadopodium formation

**Figure 3:**
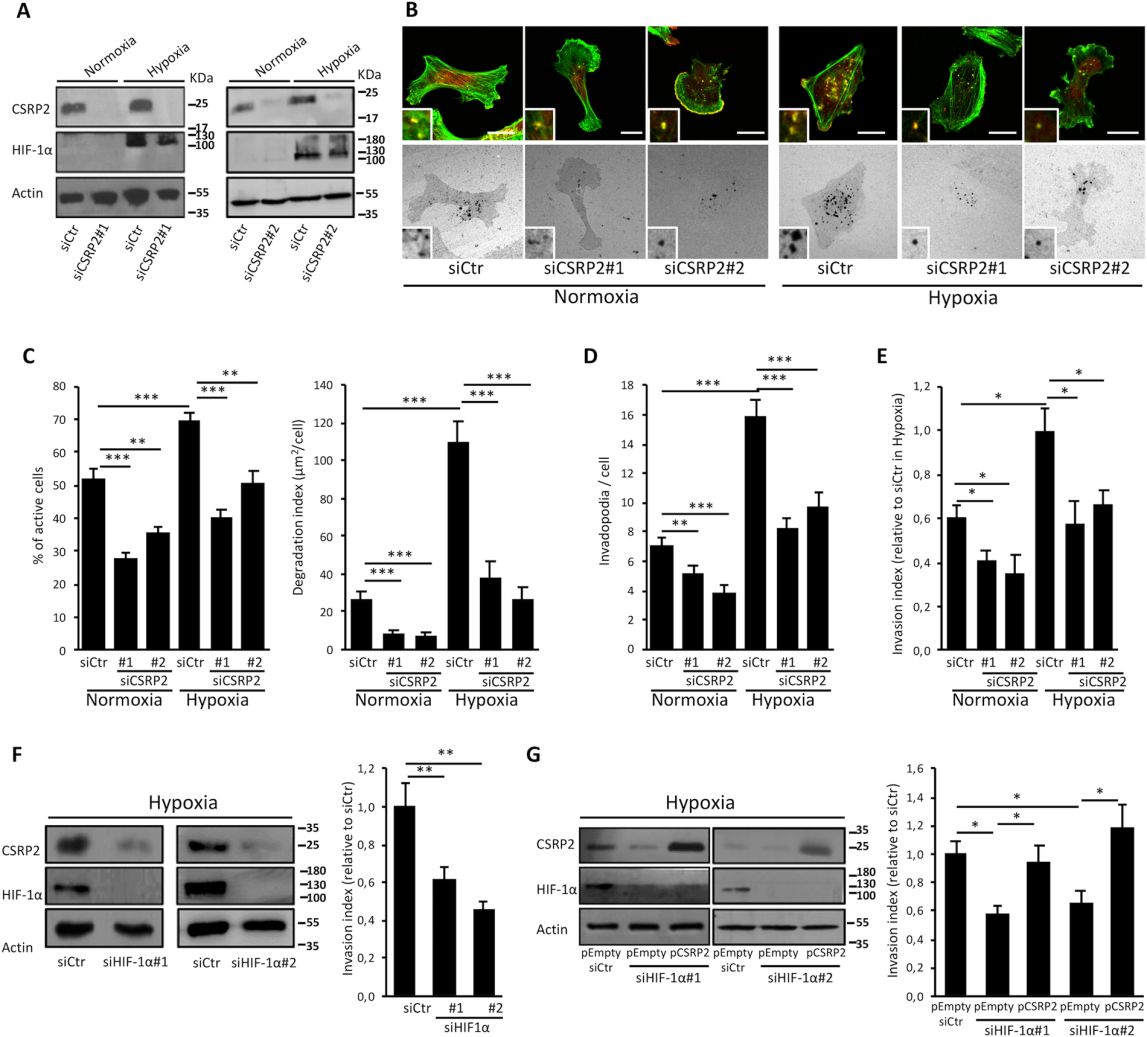
Hypoxia-dependent stimulation of MDA-MB-231 cell invasive potential is mediated by CSRP2. (**A**) Western blot analysis of CSRP2 and HIF-1α protein levels in MDA-MB-231 cells transfected with non-targeting (siCtr) or 2 different CSRP2-targeting (siCSRP2#1 and 2) siRNAs and cultured under normoxia or hypoxia. (**B**) Gelatin degradation assay. Control and CSRP2 depleted cells were plated on Oregon Green 488-labelled gelatin-coated coverslips for 16 hours, fixed and stained for actin (green) and cortactin (red, upper panels). (**C**,**D**) Quantitative analyses corresponding to experiments shown in (**B**) with actively ECM degrading cells as expressed as percentage of the total cell population (**C**) left panel), degradation index (average of degraded matrix per cell; (**C**) right panel) and the number of mature invadopodia per cell (F-actin and cortactin co-labelled puncta overlapping with areas of gelatin clearing; **D**). The data originate from at least three independent experiments (n ≥ 60 cells). (**E**) Transwell invasion assay. Invading control and CSRP2 depleted cells in normoxia and hypoxia at 24 h were quantified via MTT staining. Results were expressed relatively to the invasion of siCtr-transfected hypoxic cells (set to 1). The data originate from 3 independent experiments. (**F**) Transwell invasion assay with hypoxic MDA-MB-231 cells transfected with control (siCtr) or HIF-1α (siHIF-1α#1 and #2) targeting siRNAs. CSPR2 and HIF-1α protein levels were analysed by western blot (left panel). The data originate from 6 independent experiments. (**G**) Similar transwell invasion assay as in (**F**) with MDA-MB-231 cells that were further transfected with an empty pCDNA3.1 expression plasmid (pEmpty) or a CSRP2 overexpression vector (pCSRP2). Results were expressed relatively to the invasion of siCtr and pEmpty co-transfected cells (set to 1). The data originate from 6 independent experiments. Bars = 15 µm. *p < 0.05; **p < 0.01, ***p < 0.001.

The role of CSRP2 in hypoxia-stimulated invadopodium formation and activity was investigated in both a highly invasive cell line with significant basal levels of CSRP2 (MDA-MB-231), and a weakly invasive cell line with low basal expression of CSRP2 (MCF-7). First, fluorescent gelatin degradation assays were conducted with MDA-MB-231 cells following transfection with control scrambled siRNAs (siCtr) or CSRP2 targeting siRNAs (siCSRP2 #1 and #2; Fig. 3A). As illustrated in Figs 3B and S1, hypoxia promoted invadopodia-mediated ECM degradation in siCtr-transfected cells, as shown by a significant overall increase in the density of dark punctate in the fluorescent matrix background. Quantitative analyses revealed that the percentage cells associated with local ECM degradation (% of active cells) increased from about 50% in normoxia to about 70% in hypoxia (Fig. 3C, left panel). Even more remarkably, hypoxia induced a 5-fold increase in the average surface of matrix degradation per cell (degradation index; Fig. 3C, right panel).

Consistent with these data, siCtr-transfected MDA-MB-231 cells exhibited a higher number of mature invadopodia (as defined by F-actin and cortactin co-labelled dots overlapping with areas of gelatin clearing) and were significantly more invasive in hypoxia as compared to normoxia (Fig. 3D and E, respectively). CSRP2 knockdown inhibited the stimulatory effects of hypoxia and decreased the percentage of active cells, the degradation index and the number of invadopodia per cell to values similar to those obtained for control cells in normoxia (Figs 3B–D and S1). In addition, CSRP2 knockdown inhibited tumour cell invasion under hypoxia with an almost similar magnitude as HIF-1α knockdown (Fig. 3E and F). In contrast, CSRP2 depletion did not significantly modify the MDA-MB-231 cell migration under normoxia or hypoxia, supporting that CSRP2 has a specific function in invasion (Fig. S2). Remarkably, CSRP2 forced expression was sufficient to increases the invasiveness of HIF-1α depleted MDA-MB-231 cells under hypoxia (Fig. 3G). In agreement with our previous report^19^, CSRP2 knockdown also repressed invadopodia-mediated matrix degradation and invasion under normoxia (Fig. 3B–E). Collectively, these data suggest that hypoxia promotes invadopodia-mediated breast cancer cell invasion by upregulating CSRP2, a basic component of the invadopodial actin cytoskeleton machinery. To strengthen this conclusion, we validated the role of CSRP2 in facilitating hypoxia-stimulated invadopodia formation, ECM degradation and cell invasion in another invasive breast cancer cell line, namely the mouse 4T1 cell line (Fig. S3).

**Figure 4:**
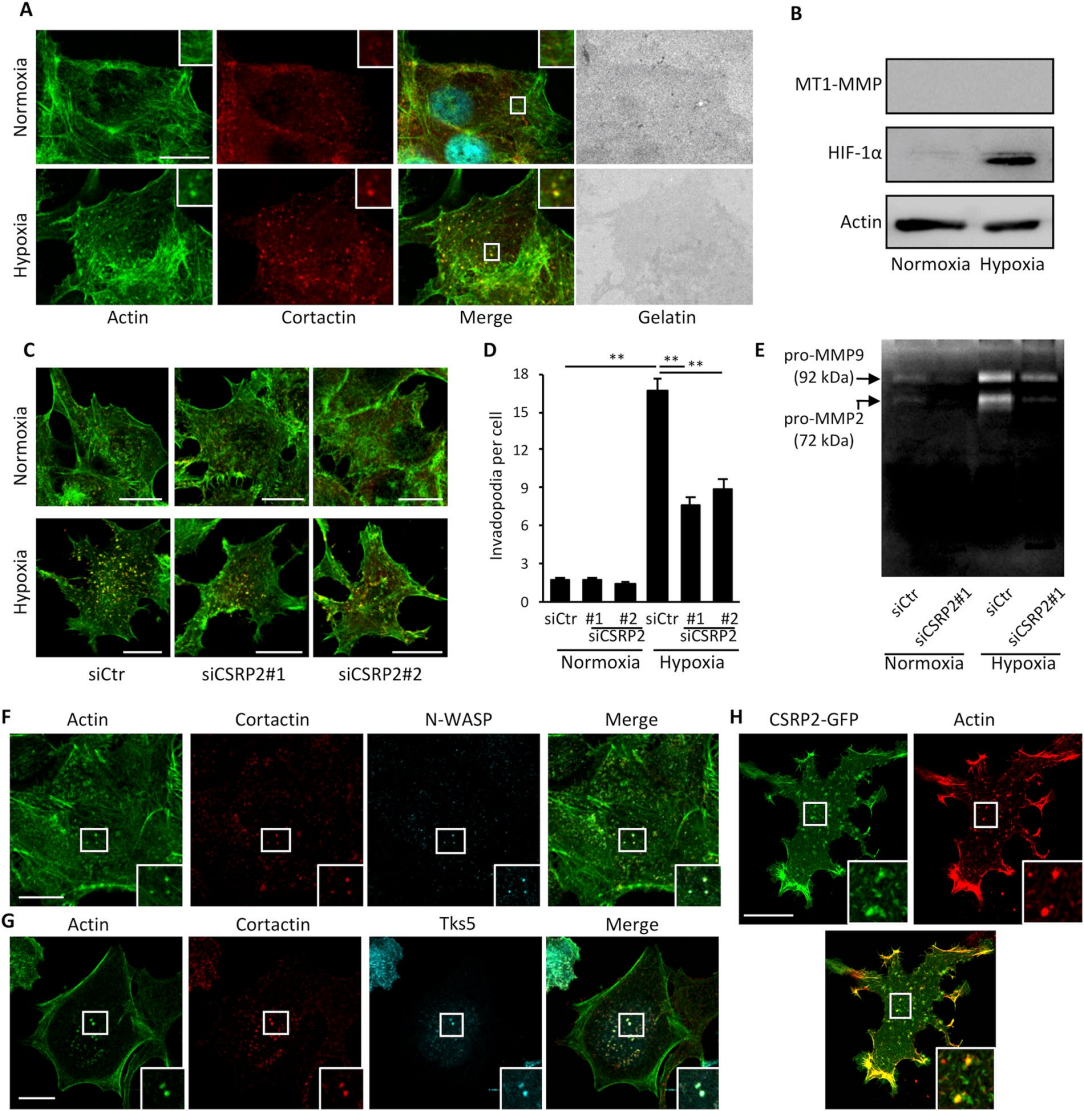
Hypoxia-induced invadopodium precursors formation in MCF-7 cells requires CSRP2 upregulation. (**A**) Gelatin degradation assay showing that normoxic (upper panels) and hypoxic (lower panels) MCF-7 cells do not promote (Oregon Green 488-labelled) gelatin degradation after 48 h. After fixation, MCF-7 cells were stained for actin (in green) and cortactin (in red), and imaged using confocal microscopy. (**B**) Western blot analysis of the MT1-MMP and HIF-1α protein levels in normoxic and hypoxic MCF-7 cells. (**C**) MCF-7 cells were transfected with non-targeting or 2 different CSRP2 siRNAs (siCSRP2#1 and 2), cultured under normoxic or hypoxic conditions, and invadopodium precursors were detected by actin (green) and cortactin (red) co-labelling. (**D**) Quantitative analysis of invadopodium precursor density in MCF-7 cells as in (**C**). The data originate from 3 independent experiments (n = 60). (**E**) Gelatin zymography assay conducted with the conditioned media collected from MCF-7 cells cultured as in (**C**,**D**). The zymogram is representative of three independent experiments. Similar data were obtained with siCSRP2#2. (**F**,**G**) Hypoxic MCF-7 cells were labelled for actin (green), cortactin (red) and either N-WASP (immunolabelling, **F**) or Tks5 (Tks5-GFP, **G**). (**H**) CSRP2-GFP co-localizes with actin (red) at invadopodium precursors in hypoxic MCF-7 cells. The insets show magnification of invadopodium precursors. Bars = 15 µm; *p < 0.05; **p < 0.01.

Contrary to MDA-MB-231 and 4T1 cells, MCF-7 cells failed to promote localized gelatin degradation under both normoxia and hypoxia (Fig. 4A). This lack of activity was consistent with the fact that MCF-7 cells did not express membrane type-1 MMP (MT1-MMP; Fig. 4B), a membrane-tethered MMP required for proteolytic activity^28,29^. However, hypoxia promoted cortactin relocalization to actin puncta, whose appearance and localization at the ventral side of tumour cells were reminiscent of invadopodia (Fig. 4A). Similar ventral actin/cortactin puncta were induced by hypoxia when MCF-7 cells were plated on non-denatured collagen (Fig. S4). To further examined the nature of these structures, we used two additional invadopodium markers, namely N-WASP and Tks5^30^. Both effectively labelled the actin/cortactin puncta that formed under hypoxia (Fig. 4F and G), which were accordingly termed invadopodium precursors. As shown in Fig. 4H, CSRP2 was also recruited to hypoxia-induced invadopodium precursors, suggesting it is required for, or possibly mediates, their formation. To assess this possibility, the number of invadopodium precursors was determined in MCF-7 cells that were transfected with control or CSRP2-targeting siRNAs prior to incubation in normoxic or hypoxic conditions. Under normoxia, both siCtr- and siCSRP2-transfected cells exhibited about two invadopodium precursors on average (Fig. 4C and D). Hypoxia increased invadopodium precursors density in control cells by almost 9-fold, with an average of 17 invadopodium precursors per cell. Interestingly, this effect was associated with a significant increase in the secretion of pro-MMP-2 and pro-MMP-9, suggesting that, although proteolytically inactive, these invadopodium precursors are mature enough for MMP secretion (Fig. 4E). CSRP2 knockdown significantly lowered the number of hypoxia-induced invadopodium precursors, with about eight precursors per cell on average (Fig. 4C and D). Accordingly, it also inhibited hypoxia-promoted secretion of proMMP-2 and proMMP-9 (Fig. 4E). Thus, although MCF-7 cells lack MT1-MMP expression and matrix degrading activity, they assemble invadopodium precursors in response to hypoxia in a CSRP2 dependent manner.

Collectively our data indicate that CSRP2 an important mediator of hypoxia-stimulated invadopodium formation in breast cancer cells.

### CSRP2 is upregulated in hypoxic, HIF-1α-positive regions of human breast cancer cell line xenografts

**Figure 5:**
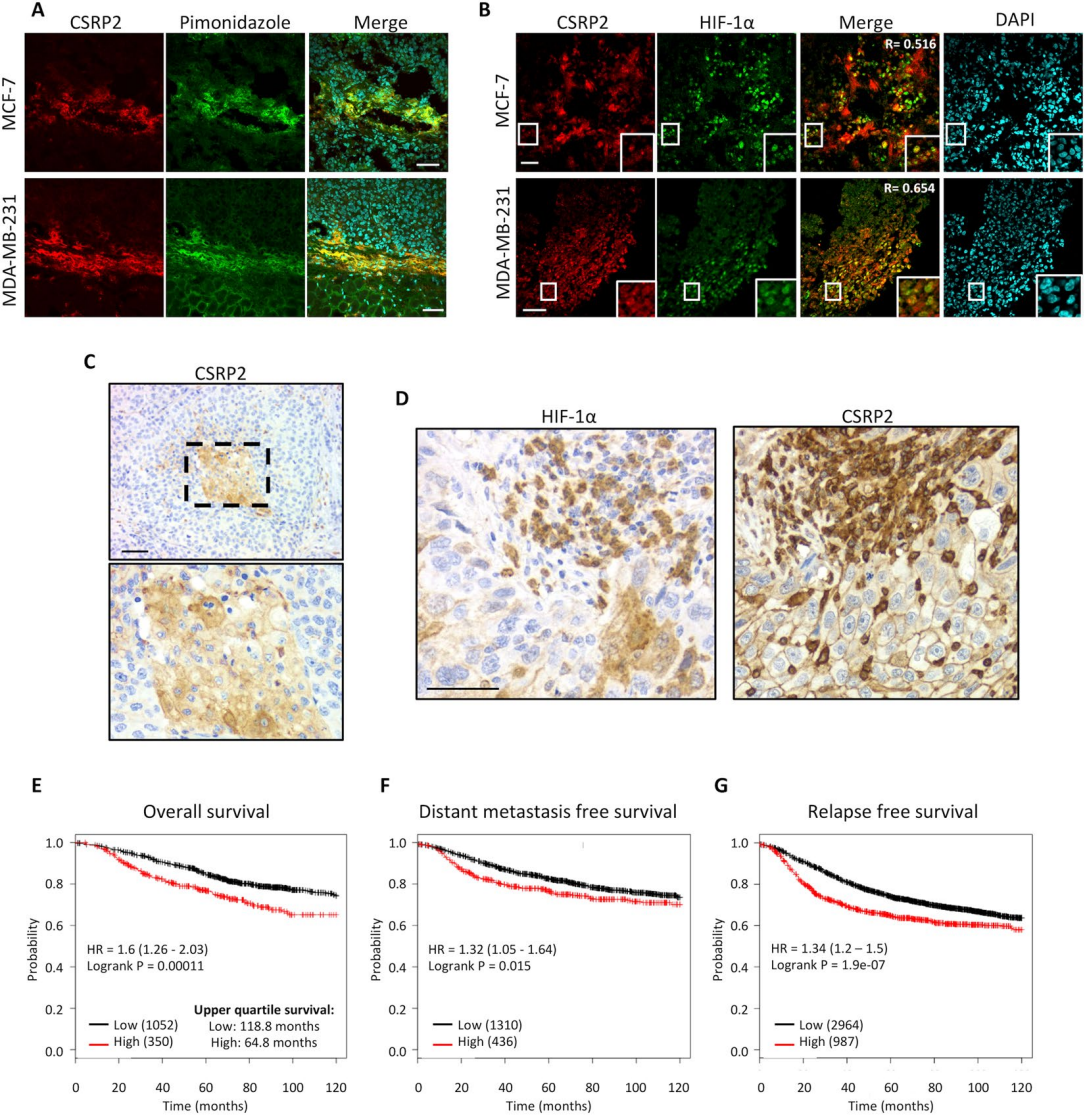
CSRP2 co-localizes with HIF-1α in tumour xenografts and human breast cancer sections, and is significantly associated with worse outcomes in breast cancer patients. (**A**,**B**) MCF-7 or MDA-MB-231 murine tumour xenografts were sectioned, stained with pimonidazole and immunolabelled for CSRP2 and/or HIF-1α. A correlation coefficient for co-localization of CSRP2 and HIF-1α is indicated in (**B**). These results are representative of at least 21 tumour sections originating from 4 animals for each type of xenograft (Fig. S5). (**C**) Immunohistochemistry showing typical CSRP2 upregulation at the centre of a tumour nest (box) in a lymph node of metastatic breast cancer. (**D**) Representative example of serial tissue sections of a triple negative breast tumour stained for HIF-1α and CSRP2 using immunohistochemistry. From the 48-sample TMA, a correlation coefficient value of 0.42 was calculated by Spearman Rank analysis (p = 0.0027; Table S1). (**E**–**G**) Kaplan-Meier survival analyses in relation to *CSRP2* expression (affy ID 207030s_at) in breast carcinoma using overall (**E**) distant metastasis free (**F**) or relapse free (**G**) survival as an endpoint. The patient samples, hazard ratio with 95% confidence interval, and *p* value (Logrank test) are displayed on each chart. Upper quartile survival for patients with low and high CSRP2 expression is indicated in (**F**). Bars = 50 µm.

To validate that CSRP2 is up-regulated by intratumoral hypoxia, MCF-7 and MDA-MB-231 cells were orthotopically injected in the mammary fat pad of immunodeficient mice, and the resulting primary tumours were collected for immunofluorescence analyses. As shown in Fig. 5A, CSRP2 was strongly upregulated in hypoxic, pimonidazole-stained, regions of both MCF-7- and MDA-MB-231-cell derived tumours. In addition, tumour sections were co-labelled for CSRP2 and HIF-1α (Fig. 5B). Strong CSRP2 signals were very frequently associated with HIF-1α positive regions in both MCF-7 and MDA-MB-231 tumour xenografts. Pixel intensity correlation analyses confirmed a strong correlation between CSRP2 and HIF-1α expression in all tumour sections analysed, with Pearson coefficient values ranging from 0.50 to 0.85 for MCF-7 cell-derived tumours, and from 0.63 to 0.86 for MDA-MB-231 cell-derived tumours (n = 22 and 21, respectively; Fig. S5). These data provide compelling evidence that CSRP2 expression is not only stimulated by experimental hypoxia produced in low-oxygen incubators, but is also induced by *in vivo* tumour hypoxia.

### Clinical significance of hypoxia-induced CSRP2 expression in breast cancer

We next assessed the co-expression of *CSRP2* and metastasis-effector genes reported to be regulated by HIF in patient tumours^31^. This analysis was conducted using a publicly available gene expression database of a large cohort of breast cancer patients (n = 1,215). We found that *CSRP2* expression positively correlates with the expression of 21 out of 25 genes for which the expression data is available (p < 0.01; Table 1). Correlation coefficient values >0.3 were obtained for 9 of these 21 genes, and correlation coefficient values >0.5 were obtained for 2 genes, *LOXL4* (*r* = 0.54; *p* = 1.*5e*-92) *and MET* (*r* = 0.50; *p* = 1.*5e*-79).

**Table 1:** Co-expression of *CSRP2* with a manually curated set of hypoxia-induced, pro-metastatic, breast cancer genes.

Gene	Correlation coefficient	p-value
*LOXL4*	0.539	1.5E-92
*MET*	0.505	1.5E-79
*COX2/PTGS2*	0.468	3.9E-67
*SNAIL1/SNAI1*	0.427	5.0E-55
*TWIST1*	0.388	5.7E-45
*ANGPTL4*	0.385	2.9E-44
*L1CAM*	0.347	1.3E-35
*LOX*	0.331	1.9E-32
*PLAUR*	0.328	5.9E-32
*MMP2*	0.303	2.8E-27
*LOXL2*	0.3	1.3E-26
*PLOD2*	0.283	7.4E-24
*PGF*	0.274	2.7E-22
*ANGPT2*	0.263	1.1E-20
*SDF-1/CXCL12*	0.255	1.7E-19
*CXCR4*	0.248	1.8E-18
*MMP14*	0.242	1.3E-17
*CXCR3*	0.18	2.6E-10
*AMF/GPI*	0.118	3.7E-05
*P4HA2*	0.097	6.9E-04
*VEGFA*	0.093	1.2E-03

A manually formulated set of 26 genes consistently and directly found to be induced by HIF-1α, as well as being drivers of invasion and/or metastasis of breast cancer cells, was analysed for co-expression with *CSRP2* in the large TCGA dataset of gene expression in invasive, human breast cancers (n = 1,215). R values were calculated using Pearson’s correlation or Spearman’s rho for those genes with a normal distribution or otherwise, respectively, and the R value was used to calculate a p-value given the *n* of the sample set.

To more directly evaluate CSRP2 up-regulation in hypoxic areas of human breast tumours, we conducted immunohistochemical analyses of clinical breast cancer specimens. Looking for hints of a hypoxic expression profile, we found that CSRP2 was frequently upregulated in or near the centre of tumour nests, a presumably highly hypoxic region (Fig. 5C). Next, we analysed and compared CSRP2 and HIF-1α protein expression in 48 triple negative breast cancer tissue array samples, and the results were scored by a pathologist (Table S1). 14/48 (>30%) cases showed weak to obvious HIF-1α expression, of which 11 (>78%) were also positive for CSRP2. Rank analysis confirmed a statistically significant correlation between CSRP2 and HIF-1α protein levels in clinical samples with a correlation coefficient value of 0.42 (p = 0.0027). In addition, CSRP2 and HIF-1α exhibited partially overlapping distribution in successive tumour sections (Fig. 5D). As previously reported^19^, CSRP2 staining was also frequently observed in inflammatory/immune cells. Such staining likely account for the lower, yet significant, correlation obtained for clinical samples as compared to mouse xenografts.

To evaluate the clinical relevance of our findings, the prognostic value of CSRP2 in breast cancer was evaluated by Kaplan Meier analysis using transcriptomics data sets including 1402 breast cancer patients^32^. High *CSRP2* mRNA expression was significantly associated with reduced overall survival for breast cancer patients. The best cut off between low and high *CSRP2* expression to discriminate the subgroups with different outcomes was determined to be the upper quartile (HR = 1.6, logrank P = 0.00011; Fig. 5E). Upper quartile survival, i.e. time beyond which 75% of the patients are expected to survive, for patients with low and high *CSRP2* expression was 118.8 and 64.8 months, respectively. Stratifying patients according to upper tertile or median *CSRP2* expression values established weaker, yet significant, association between *CSRP2* expression and overall survival (HR = 1.49, logrank P = 0.00056, and HR = 1.28, log-rank p = 0.025, respectively; Fig. S6). In addition to overall survival, CSRP2 overexpression significantly correlated with shorter distant metastasis- and relapse- free survival (p = 0.015 and 1.9e-07, respectively; Fig. 5F and G). Comparison of *CSRP2* expression in different breast cancer subtypes revealed that *CSRP2* expression is an order of magnitude higher in the basal subtype, compared to the other subtypes and, accordingly, is a stronger predictor of distant metastasis-free survival (Fig. S7).

Taken all together, our data suggest that tumour hypoxia promotes CSRP2 overexpression, exacerbating the clinical outcome of breast cancer patients by enhancing tumour cell invasion and subsequent spread to distant tissues.

## Discussion

Both experimental and clinical studies have provided evidence that the hypoxic tumour microenvironment is an important risk factor for distant metastasis in breast cancer^3^. Here, we identified a novel mechanism by which hypoxia promotes mesenchymal invasion in breast cancer cells through the upregulation of CSRP2, a structural component of the actin cytoskeleton machinery involved in invadopodium formation^19^. Our functional investigations revealed that CSRP2 knockdown in MDA-MB-231 cells inhibits hypoxia-stimulated invadopodia formation and ECM degradation. Accordingly, it also significantly suppressed the stimulatory effect of hypoxia on MDA-MB-231 cell invasiveness. Conversely, forced CSRP2 expression in HIF-1α-depleted hypoxic MDA-MB-231 cells was sufficient to increase cell invasion to an extent similar as in control hypoxic cells. Together these data support that CSRP2 is not only required for hypoxia-stimulated invasion, but contributes to drive this process.

Contrary to MDA-MB-231 cells, MCF-7 cells were unable to carry out localized gelatin matrix degradation under normoxia or hypoxia. In keeping with this, our western blot analyses revealed that both normoxic and hypoxic MCF-7 cells do not express MT1-MMP, a membrane anchored MMP which is critically required for tumour cell proteolytic activity^28,29^. Although proteolytically inactive, MCF-7 cells responded to hypoxia by assembling invadopodium precursors characterized by the re localization of cortactin, N-WASP and Tks5 to actin accumulations at the ventral cell membrane. This finding is highly consistent with a previous report showing that MT1-MMP depletion in MDA-MB-231 cells strongly inhibits gelatin matrix degradation but only modestly alters the onset of invadopodium formation^29^. In tumour cells, soluble MMP secretion is facilitated at invadopodia^33^. Thus, the increased secretion of MMP-2 and MMP-9 that is associated with the formation of invadopodium precursors in hypoxic MCF-7 cells (Fig. 4E) represents another piece of evidence of the nature of these structures. Consistent with the fact that MT1-MMP catalyses MMP-2 activation by cleavage of its pro-domain^34^, and that MMP-2 contributes to MMP-9 activation^35,36^, mostly inactive, high-molecular weight, forms of MMP-2 and MMP-9 were detected in our serum-free gelatin zymography assays.

Since invadopodium assembly and activity seems to be decoupled in hypoxic MCF-7 cells, this cell line turned out to be an appropriate experimental model to further characterize the mechanism by which CSRP2 mediates hypoxia-stimulated invasion. Indeed, our quantitative data show that CSRP2 knockdown significantly reduced the number of invadopodium precursors induced by hypoxia in MCF-7 cells, validating the structural role of CSPR2 in invadopodium assembly we previously suggested^19^.

Our mechanistic investigations revealed that the proximal promoter of CSRP2 can be activated by either hypoxic culture conditions or HIF-1α overexpression, and that it contains two HREs (HRE1 and 2) able to promote HIF-1 recruitment. Although both HREs were found to contribute to hypoxia-induced transactivation of *CSRP2* promoter, our data luciferase reporter assays suggest that HRE2 has a predominant role over HRE1. Consistent with ChIP and luciferase reporter data, CSRP2 and HIF-1α protein levels were significantly correlated in both pre-clinical and patient tumour specimens, supporting that tumour hypoxia is an important determinant of CSRP2 up-regulation in breast cancer. The four breast cancer cell lines we analyzed responded to experimental hypoxia by increasing CSRP2 mRNA and protein expression. As previously reported^19^, invasive cells (MDA-MB-231 and Hs578T) exhibited significant basal CSRP2 levels under normoxia while weakly invasive cells (T47D and MCF7) did not. We noticed that invasive cells frequently exhibited detectable amounts of HIF-1α under normoxia (as evaluated by western blot, Fig. 1A and B). Normoxic stabilization of HIF-1α and activation of HIF-1 signalling in triple negative breast cancer cells, such as MDA-MB-231 cells, was recently shown to be regulated by a long noncoding RNA, namely *LINK-A*^37^. This pathway may account, at least to some extent, for normoxic expression of CSRP2 in triple negative breast cancer cells, which in turn contributes to maintain a constitutive invasive phenotype^19^. The significant basal expression level of CSRP2 in invasive normoxic cells (Fig. 1) is not contradictory with the role of CSRP2 in mediating the pro-invasive effects of hypoxia, and shows that hypoxia operates in breast cancer cells by up-regulating an actin regulatory protein that is also required for normoxic invasion. Other hypoxia-invadopodia axes were identified in cancer of different origins, such as melanoma, fibrosarcoma, and head and neck cells^38–41^. To our best knowledge, our report is the first example where hypoxia promotes cancer cell invasion by direct, HIF1-mediated, targeting of a basic structural cytoskeletal component of invadopodia.

A recent study has provided evidence that pancreatic ductal adenocarcinoma (PDAC) invasion and metastasis are promoted by HIF-1 dependent upregulation of another actin bundling protein, namely fascin^42^. Although the effects of fascin overexpression on PDAC cell invadopodium formation were not specifically evaluated, fascin has been shown to be a critical invadopodial component in prostate cancer and breast cancer cells^16,43,44^. Noticeably, fascin and CSRP2 knockdown achieves comparable reduction in ECM degradation and 3-D invasion in invasive breast cancer cells^16,19,43^, suggesting a functional interaction/redundancy between these two actin bundling proteins. Remarkably, fascin ranks first in the most highly correlated genes to *CSRP2* in invasive breast cancer tumours (publicly available TCGA data at the cBioPortal for Cancer Genomics, http://www.cbioportal.org), raising the possibility that hypoxia and HIF-1 coordinate CSRP2 and fascin upregulation to promote invadopodium assembly/stabilization and invasion.

The clinical significance of our findings is further supported by transcritomic-associated survival analysis indicating that CSRP2 overexpression is associated with significantly shorter overall, metastasis- and relapse-free survival. Besides its role in breast cancer, CSRP2 was recently identified as a downstream target of *H19*, the long non-coding RNA with the strongest association with colorectal cancer patient survival^45^. Patients with high CSRP2 expression in colorectal tumours, display significantly shorter overall survival, and combined analysis of *H19* with *CSRP2* appears to be a powerful prognostic factor for overall survival. Another recent clinical study established a significant association between CSRP2 expression and B-cell acute lymphoblastic leukemia (ALL) relapse, and proposed CSRP2 as a prognostic marker for B-cell ALL patients with normal cytogenetics^46^. Functional analyses suggest that CSRP2 promotes B-cell ALL cell proliferation, migration, and drug resistance. Because hypoxia is a common feature of the bone marrow microenvironment that promotes HIF-dependent blood cancer progression and resistance to therapy^47^, our data call for evaluation of hypoxia-regulated CSRP2 expression in blood cancers. Although the specific functions of CSRP2 in breast cancer, colorectal cancer and B-cell ALL may differ, these studies all point to the deleterious clinical consequence of CSRP2 overexpression.

Collectively, our data indicate that hypoxia exerts a direct control on invadopodium-mediated tumour cell invasion through HIF-1-mediated upregulation of the actin-bundling protein CSRP2 and provide a new mechanistic basis for the pro-metastatic effects of tumour hypoxia in breast cancer.

## Methods

### Cell lines

MCF-7 cells were purchased from ATCC. MDA-MB-231, Hs578T, T47D and 4T1 cells were available at the Luxembourg Institute of Health. These cell lines were regularly tested for mycoplasma contamination and cultured in complete growth medium following ATCC recommendations. A standard tissue culture incubator was used for a normoxic culture conditions (21% O_2_; 5% CO_2_). For hypoxic treatment, cells were placed in a hypoxia work station (Invivo2 400, Ruskinn) for 24 hours or 48 hours, calibrated to maintain a hypoxic atmosphere of 0.1% O_2_ and 5% CO_2_ by continuous flow of nitrogen. The CSRP2-depleted 4T1 and corresponding control cell lines by lentiviral transduction. CRP2 knockdown was achieved by pGIPZ lentiviral shRNAs (clone ID: V3LMM_417823 from GE Dharmacon, gene set RMM4532). A non-silencing shRNAs (RHS4346, sh-; GE Dharmacon) was used as a control. Lentivirus production was achieved by co-transfecting lentiviral pGIPZ shRNAs with packaging and envelope plasmids in HEK293T cells using Xtreme transfection reagent (Roche). 4T1 cells were infected with virus, and transduced cells were selected with 0.5 μg/ml puromycin (Sigma-Aldrich).

### Western blot analysis

Total protein extracts from cells was prepared in RIPA lysis buffer (Millipore) supplemented with protease and phosphatase inhibitor mixture (Roche). The extracts were subjected to Western blot using antibodies directed against CSRP2 (HPA045617, Sigma), Actin (A2066, Sigma), HIF-1α (610959, BD biosciences), and MT1-MMP (ab51074, Abcam. Protein bands were detected using Western Lightning Ultra (Perkin Elmer) and visualized with CL-Xposure film (Thermo Scientific). Protein levels were quantified using ImageJ (NIH, Bethesda, USA).

### siRNA transfection

CSRP2 knockdown was achieved by transfection of 10 nM of 2 different predesigned siRNAs directed against human CSRP2 (siCSRP2#1, SI04283727, target sequence 5′-ACAGTGGCAATTCACGATGAA-3′ and siCSRP2#2, SI04251863, target sequence 5′-ACAGGCCTACAACAAATCCAA-3′; QIAGEN) using DharmaFECT^TM^ transfection reagent (GE Dharmacon) and following manufacturer’s instructions. HIF-1α knockdown was achieved using 2 different predesigned, functionally verified, siRNAs directed against human HIF-1α (siHIF1#1, SI02664053, targeted sequence 5′-AGGAAGAACTATGAACATAAA-3′, Qiagen and siHIF1#2, targeted sequence 5′-TACGTTGTGAGTGGTATTATT; Eurogentec), while non-targeting siRNAs were used as a control (Eurogentec). Freshly transfected cells were incubated 24 h under normoxia prior subsequent treatment or analysis.

### Quantitative RT-PCR

Total RNA was isolated using the miRCURY RNA Isolation Kit (Exiqon) following the manufacturer’s instructions. Purified RNA was reverse transcribed to cDNA using the Reverse Transcriptase Core kit (Eurogentec). Quantitative real-time PCR reactions were performed using SYBR Green I (Qiagen) on an Applied Biosystems ViiA 7 Real-Time PCR System (Thermo Fisher Scientific). The primers used were 5′-GATCTCGGACTCCCTGGAC-3′ (forward) and 5′-TCCCCAGACAGGCATTTT-3′ (reverse) for CSRP2 and 5′-TGTCCTGAATGTGGTCACCTGA-3′ (forward) and 5′-CTGCAGTCTCCTTGCACACCT-3′ (reverse) for mitochondrial ribosomal protein L32 (MRPL32). To calculate the relative abundance of the mRNA transcripts, the ΔΔCt method was used, with MRPL32 used as a reference mRNA. The Y-axis reflects fold change in gene expression.

### Chromatin immunoprecipitation assay

MCF-7 cells were grown in 15 cm plates. Once the cells had reached 70% confluence, they were subjected to 24 hours of normoxia or hypoxia, as indicated. Chromatin was isolated and sheared using the Bioruptor Pico sonicator (Diagenode), with 10 cycles of 30 seconds on at high power, followed by 30 seconds off. Sheared chromatin was used for ChIP, with anti-HIF-1Αα (ChIP grade, Active Motif, 39665) or negative control antibodies, with the ChIP-IT Express kit (Active Motif), according to the manufacturer’s protocol. Pulled-down chromatin was then purified using the Chromatin IP DNA Purification Kit (Active Motif) and RT-PCR was performed using the following primer pairs: 5′-AAGTCCCTCTCCAAGTCC-3′ (forward) and 5′-CCACCAGGAGACAAAAGG-3′ (reverse) for HRE1, and 5′-TGCCTTTTGTCTCCTGGTG-3′ and 5′-ATGGGGATGTCGGAGGAGA-3′ (forward) for HRE2.

### Luciferase reporter assay

Genomic DNA was purified from MCF-7 cells, using the AllPrep DNA/RNA/Protein Mini Kit (Qiagen). A region corresponding to −1,920 to +103 bp relative to the transcription start site of the human *CSRP2* gene was PCR amplified using the primers 5′-GATCACGCGTCAGGGACAGACAGCAGAAACA-3′ (forward) and 5′-GATCAGATCTTTGAGTCGGAGGCGGGAGCACGTAC-3′ (reverse), and inserted into the*MluI-BglII* multiple cloning site of the pGL3-Promoter vector (Promega). The HRE1 and HRE2 sites were mutated using overlapping PCR strategy and the following primer pairs: 5′-GAGGGAGCGGTCCCGGGAGCTGGGAAG-3′ (forward) and 5′-CGCCCGGCATCTTCCCAGCTCCCGG-3′ (reverse) for HRE1*, and 5′-CTAGCTCCGCCTGCGGTAGCTGCTCC-3′ (forward) and 5′-CTCACTTGAGTCGGAGGCGGGAGCAGCTACCGC-3′ (reverse) for HRE2*. The VEGF promoter luciferase reporter plasmid was provided by Dr. Amato J. Giaccia (Standford University school of Medicine, CA, USA). Briefly, 7 × 10^4^ cells were seeded in each well of a 24-well plate. Six hours after plating, cells were transfected with the promoter luciferase reporter constructs, pGL4.73 [hRluc/SV40] vector (which contains the Renilla luciferase sequence downstream of the SV40 promotor), and, where indicated, a constitutively stable mutant HIF-1α expression vector (Addgene ref #52636), using Turbofect (Thermo Fisher Scientific), according to the manufacturer’s protocol. Immediately after transfection, the plates were placed in either a normoxic or hypoxic incubator, as indicated. Twenty four hours after transfection, firefly and Renilla luciferase activities were measured using the Dual-Luciferase Reporter assay kit (Promega) and the ratio of firefly/Renilla luciferase was determined.

### Cell invasion and migration assays

CSRP2- or HIF-1-depleted cells and control cells were incubated for 24 hours in hypoxia or normoxia. Fifty thousand cells were subsequently transferred onto 8.0 µm-pore transwell inserts (Greiner) in serum free DMEM, as previously described^19^. For invasion assays, transwell inserts were pre-coated with 100 µl of EHS matrix (330 µg/ml in serum-free DMEM) and incubated for 1 hour at 37 °C to allow polymerization. For migration assays, transwell inserts were pre-coated with collagen (100 µg/ml, collagen I, Millipore). The wells were filled with DMEM supplemented to 10% FBS as a chemoattractant. After 18 hours (MDA-MB-231 cell migration), 24 hours (MDA-MB-231 cell invasion) or 48 hours (4T1 cell invasion) incubation in hypoxia or normoxia, the total number of cells and the number of invasive cells were evaluated by MTT, and the percentage of invasion was calculated and normalized to siCtr-transfected normoxic cells (set to 1). For some specific assays, cells were co- transfected with HIF-1A targeting siRNAs and a plasmid allowing CSRP2 overexpression. The latter plasmid was generated by inserting CSRP2 coding sequence into the XhoI-BamHI cloning site of modified pCDNA 3.1- (Invitrogen) including a terminal HA tag. The primers used to PCR amplify CSRP2 coding sequence were 5′-GATCCTCGAGATGCCTGTCTGGGGAGGTGG-3′ (forward) and 5′-GATCAGGATCCCGCTGGGCATGAACAAGAGCCC-3′ (reverse). An empty pCDNA 3.1- was used as a control.

### Gelatinase zymography assays

Four hundred thousand MCF-7 cells were plated in each well of a six-well plate. Six hours later, they were transfected with either control or CSRP2-targeting siRNAs. Twenty-four hours later, cells were washed with PBS, incubated with serum-free DMEM, and the plates were incubated in normoxic or hypoxic conditions for 32 hours. The conditioned media was collected and concentrated using Amicon Ultra Centrifugal Filters, 30 kDa cut-off (Merck Millipore, 803024), from an initial volume of two ml to a final volume of 22 µl. The conditioned media was then subjected to gelatinase zymography analysis. Briefly, the conditioned media was mixed with 4x sample buffer (0.25 Tris/HCl pH 6.8, 40% glycerol, 8% SDS and 0.1% bromophenol blue) and resolved on a 10% SDS-polyacrylamide gel containing 0.01% porcine gelatin (Sigma, G-8150). The MMPs were then renatured by soaking the gel in renaturing buffer (2.5% Triton in dH_2_O) for 30 minutes. The gels were then washed several times with dH_2_O and incubated for 30 minutes with developing buffer (0.05 M Tris-HCl pH 7.8, 0.2 M NaCl, 0.005 CaCl_2_, and 0.02% Brij-35). The buffer was then poured out, fresh buffer added and the gel was incubated overnight at 37 C. The following day, the gels were visualized with Coomassie blue staining.

### Fluorescent gelatin degradation assays

Fluorescent gelatin slides were prepared similarly as described in Artym *et al*.^29^. In brief, 16-mm coverslips were coated with poly-L-Lysine (25 µg/ml, 20 minutes at room temperature) and crosslinked with glutaraldehyde (0.5%, 15 minutes at room temperature). After extensive PBS washing, coverslips were inverted on a 60 µl drop of 0.2% gelatin solution containing a mix of 1:20 Oregon green 488-labelled gelatin (Molecular Probes) and unlabeled porcine gelatin (Sigma). After a 15-minute incubation, and 3 washes, coverslips were sterilized with 70% ethanol for 30 minutes and subsequently incubated with complete medium for 30 minutes. MDA-MB-231 cells were pre-incubated for 32 hours in hypoxia or normoxia before they were transferred onto gelatin-coated coverslips for a 16-hour incubation period in the same culture conditions. A low-density seeding was used to avoid cell clustering and facilitate the analysis. MCF-7 and 4T1 cells were directly loaded onto gelatin-coated coverslips and incubated for 48 hours in hypoxia or normoxia. Then, tumour cells were fixed and stained for actin and cortactin (see “Confocal microscopy and cell imaging” subsection). Active cells were defined as cells with dark dots (corresponding to fluorescent gelatin-cleared areas) underneath actin-cortactin co-labelled invadopodia. Digested gelatin areas were quantified with the threshold tool in ImageJ software, and an average degradation index (degraded matrix area per cell) was calculated.

### Confocal microscopy and cell imaging

Imaging was performed on a laser scanning confocal microscope (LSM880 FastAiry, Carl Zeiss) equipped with a x63/1.4 numerical aperture (NA) oil immersion Plan-Apochromat objective for cell imaging or a x40/1.3 oil immersion Plan-Apochromat for tumour slice imaging. All pictures were acquired with multitrack configuration with a confocal optical slice set at 1 µm thickness. MDA-MB-231, 4T1 and MCF-7 cells were plated on gelatin-coated coverslips (see “Fluorescent gelatin degradation assays” subsection) for 16 hours or 48 hours. MCF-7 cells were also plated on collagen-coated coverslips (100 µg/ml, collagen I, Millipore) for 48 hours. Pre-fixation was performed in 3.5% PFA and 0.3% Triton X-100 in PEM (100 mM PiPES, pH6.9, 1 mM EGTA and 1 mM MgCl_2_) for 3 minutes whereas fixation was performed in PFA 3.5% in PEM for 20 minutes. Cortactin and N-WASP were immunodetected using mouse monoclonal antibodies (clone 4F11, Millipore) and rabbit monoclonal antibodies (clone 30D10, Cell Signalling), respectively. The actin cytoskeleton was labelled using Acti-stain 488 or 555 or 670 (Cytoskeleton) phalloidin depending on the experimental conditions. To facilitate evaluation, actin cytoskeleton was always depicted in green regardless of the type of staining used. Before confocal microscopy imaging, coverslips were mounted in Fluoromount medium (Sigma). In some experiments, MCF-7 cells were transfected using Lipofectamine 2000 (Thermo Fisher Scientific) with a construct coding for GFP-fused Tks5 (Tks5-GFP; kind gift from Dr. Sara A. Courtneidge, Portland, USA) or a construct coding for GFP-fused CSRP2 (CSRP2-GFP). The latter was obtained by inserting the CSRP2 coding sequence into the pEGFP-N1 plasmid vector *via* XhoI and BamHI restriction sites. After 48 hours in hypoxic conditions, MCF7 cells expressing CSRP2-GFP were incubated 4 hours with live cell imaging actin probe (siR-Actin 1 µM, Cytoskeleton) before observation under the microscope in hypoxic conditions (5% CO_2_ and 0.1% O_2_).

### *In vivo* xenograft assays and immunofluorescent staining

Animal work was conducted in accordance with the national and international regulations. The protocols were reviewed and approved by the animal welfare body of LIH (protocol LECR-2016-07), and received an authorization from the Ministry of Agriculture and Ministry of Higher Education and Research. Five million of MCF-7 or MDA-MB-231 tumour cells in 50 µl matrigel diluted 1:1 in PBS were subcutaneously implanted into the mammary fat pad of NOD *scid* gamma (NSG; NOD.Cg-Prkdc^scid^ Il2rg^tm1WjI^/SzJ), 8-week-old female mice (Charles River). Primary tumour dimensions (length, width and height) were periodically measured using calipers and the tumour volume was calculated according to the formula: ½ × L × W × H. When tumours reached a size of approximately 200 mm^3^, they were harvested and flash frozen in isopentane.

The tumours were sectioned with a cryostat (Leica SM1850 UV) in 120 µm thick sections and fixed in 4% paraformaldehyde for 15 min. Then sections were permeabilized for 15 min in 0.1% Triton followed by 3 baths in 0.1% NaBH4. HIF-1α and CSRP2 were immunolocalized using mouse monoclonal (610959, BD Biosciences) and rabbit polyclonal antibodies (HPA045617, Sigma), respectively. For correlation analyses, sections were fixed in acetone:chloroform (1:1) for 5 min at −20 °C, which results in a better preservation of CSRP2. HIF-1 and CSRP2 co-localization was estimated by calculating Pearson’s coefficient values using the JACoP plugin from ImageJ software^48^. Twenty-one and 22 sections originating from 4 independent MCF-7 and MDA-MB-231 xenograft tumours, respectively, were used for calculations. Scattered plots were generated using the Zen 2.1. software of the confocal microscope (LSM880 FastAiry, Carl Zeiss). Staining of tumour hypoxic regions was achieved using The Hydroxyprobe^TM^-1 Plus Kit (Hydroxyprobe hpi, Burlington, MA, USA). Mice were injected intraperitoneally with a pimonidazole HCL solution at a dosage of 60 mg/kg body weight 30 minutes prior sacrifice. FITC-conjugated IgG1 mouse monoclonal antibody from the same Kit was used for immunofluorescence staining.

### Immunohistochemistry

Human triple negative breast cancer tissue arrays (BRC964) including 48 cases were purchased from Pantomics, Inc. (Richmond, CA, USA). Immunohistochemical staining was performed as previously described^19^ using rabbit polyclonal antibodies against human CSRP2 (HPA045617, Sigma-Aldrich; 1/250) and rabbit monoclonal antibodies against HIF-1α (EP118; Epitomics). Rabbit anti-cytokeratin and normal rabbit serum were used as positive and negative controls. The results were semi-quantitatively scored by a pathologist as followed: 0: no signal, 0.5: insufficient signal, 1: weak signal, 2: moderate signal, and 3: strong signal. Values below 1 were considered as negative. For statistical analyses, an intensity index was calculated by multiplying the intensity score by the percentage of positive cells. Intensity index values were used to rank all the samples for Spearman rank correlation test.

### Statistical analysis

All numerical data are shown as mean ± SEM. Error bars represent standard errors. Statistical significance was determined by Spearman rank correlation test for the *in vivo* immunohistochemical results, paired two-tailed Student’s t distribution test for transwell cell invasion assay, and unpaired two-tailed Student’s t test for the other analyses. For the analysis of the active cell population on gelatin, p values were calculated from the Z-score for 2 population proportion. P values < 0.05 were considered statistically significant.

The expression analysis of HIF-1α target genes and of *CSRP2* in human breast cancer here is wholly based upon data generated by the TCGA Research network: http://cancergenome.nih.gov/. The TCGA breast cancer gene expression data was downloaded from the UCSC Cancer Genome Browser. Normality of the distribution of expression values for each gene was assessed using the D’Agostino-Pearson omnibus test. *CSRP2* expression was normally distributed, so for genes that had a normal expression distribution, a Pearson’s correlation coefficient was calculated. For genes that were not normally distributed, a Spearman’s rank correlation coefficient was calculated. These values were then used to calculate a p-value, given the *n* of the sample size (1,215 in this case). To compare *CSRP2* across different breast cancer subtypes, one-way ANOVA was used to confirm that the groups were statistically significantly different and two-tailed t-tests were performed to compare the groups in a pair-wise fashion.

Kaplan-Meier survival plots, hazard ratio with 95% confidence intervals and log-rank p values were generated using the Kaplan-Meier Plotter tool^32^ to test for associations between CSRP2 expression (Affy probe 207030s_at), and overall-, metastasis- and relapse-free survival in breast cancer patients. Gene expression data and survival information were downloaded from GEO, EGA and TCGA. Only Affymetrix HG-U133A, HG-U133 Plus 2.0 and HG-U133A 2.0 arrays were included. Biased arrays were excluded and redundant samples were removed.

## Electronic supplementary material

Supplementary Fig. S1-S7 with legends

Supplementary Table S1
