# Supplementary material for: Hypoxia promotes breast cancer cell invasion through HIF-1α-mediated up-regulation of the invadopodial actin bundling protein CSRP2
Source: Sci Rep. 2018 Jul 5;8:10191. doi: 10.1038/s41598-018-28637-x (PMC6033879; doi:10.1038/s41598-018-28637-x)
Supplement: Supplementary file 1 — Supplementary Fig. S1-S7 with legends [file 41598_2018_28637_MOESM1_ESM.pdf]

Hypoxia promotes breast cancer cell invasion through HIF-1α-mediated up-regulation of the invadopodial actin bundling protein CSRP2

Céline Hoffmann[1]†, Xianqing Mao[1]†, Joshua Brown-Clay[1], Flora Moreau[1], Antoun Al Absi[1], Hannah Wurzer[1], Barbara Sousa[2], Fernando Schmitt[2], Guy Berchem[1], Bassam Janji[1] and Clément Thomas[1]*

[1]Laboratory of Experimental Cancer Research, 84 Val Fleuri, L-1526 Luxembourg.

[2]IPATIMUP- Institute of Molecular Pathology and Immunology of the University of Porto. Medical Faculty of Porto University, Rua Julio Amaral de Carvalho 45, 4200-135, Porto, Portugal.

Supplementary Figures S1-S7.

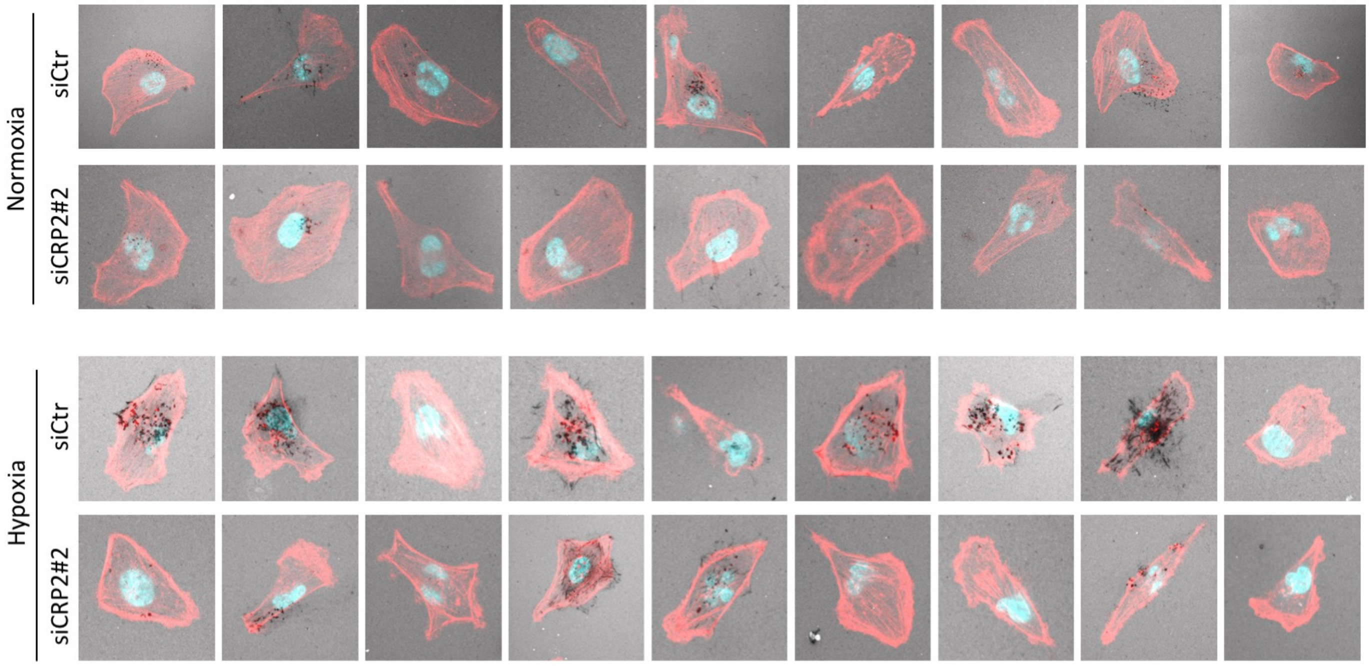

Fig S1. CSRP2 knockdown inhibits hypoxia promoted invadopodia-mediated ECM degradation MDA-MB-231. Fluorescent gelatin degradation assay in normoxic or hypoxic conditions. Random pictures from one experiment with control (siCtr) and CSRP2-depleted (siCRP2#2) cells plated on Oregon Green 488-labelled gelatin-coated coverslips for 16 hours, fixed and stained for the actin cytoskeleton (red) and the nucleus. These are examples of pictures used for the quantitative analyses shown in Fig. 3C.

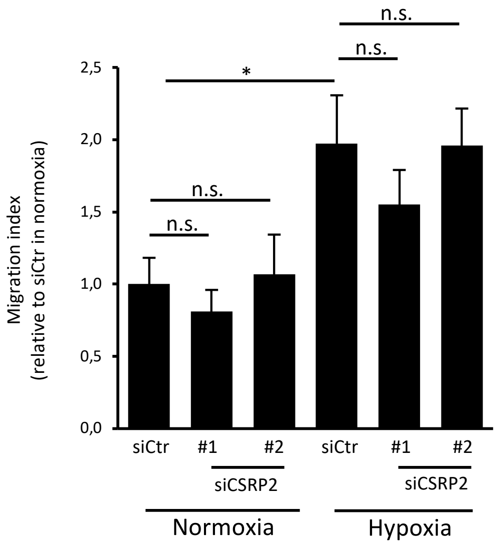

Fig. S2. CSRP2 is not required for MDA-MB-231 cell migration under normoxia or hypoxia. Transwell migration assay with normoxic or hypoxic MDA-MB-231 cells transfected with control (siCtr) or two different CSRP2-targeting siRNAs (siCSRP2 #1 and 2). Cells were plated on collagen-coated transwell for 18h and quantified via MTT staining. Results were expressed relatively to the migration of siCtr-transfected normoxic cells (set to 1). The data originate from 3 independent experiments. $*p < 0.05$.

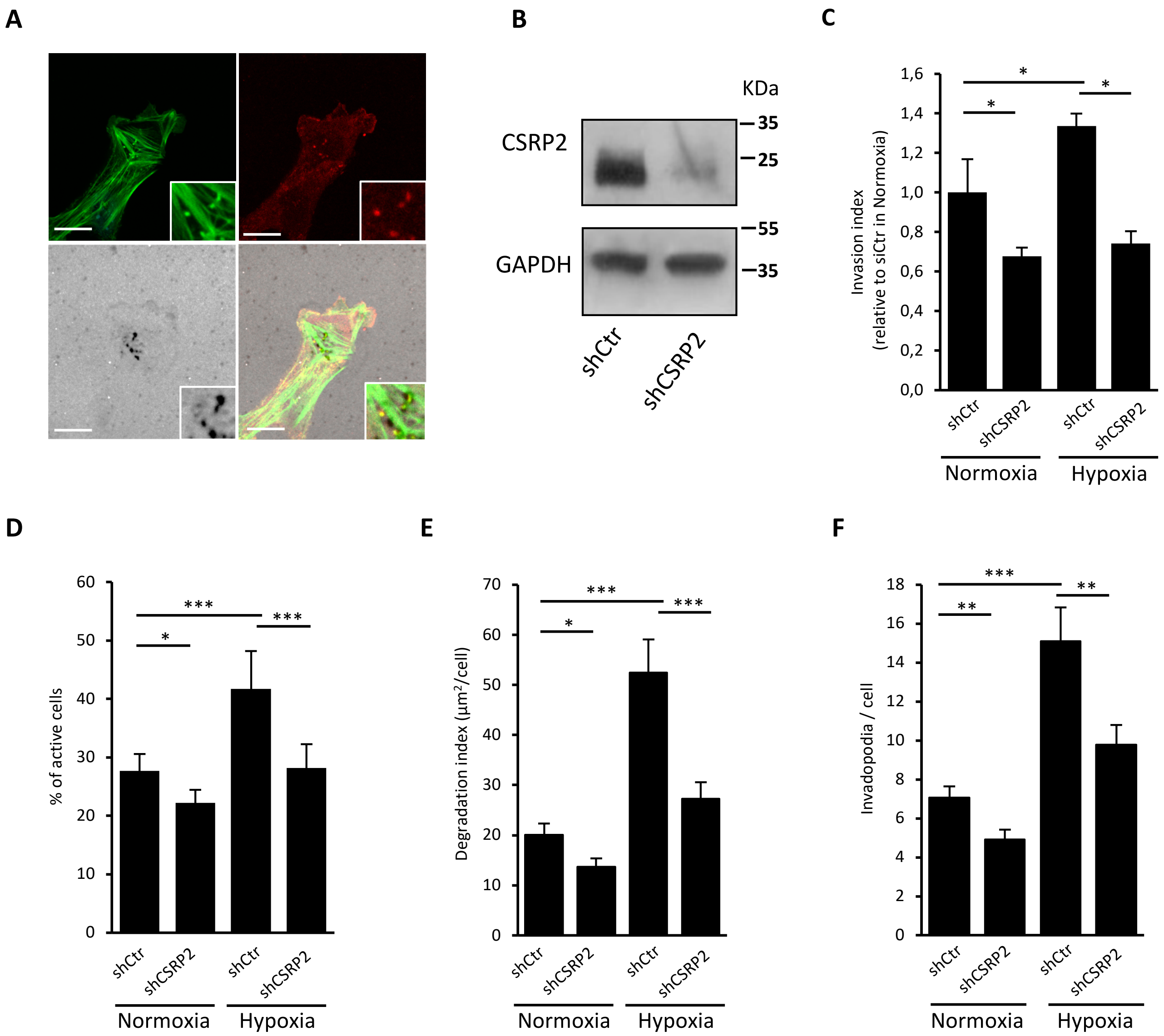

Fig. S3. CSRP2 is required for hypoxia-stimulated invadopodia formation, ECM degradation and cell invasion in mouse 4T1 cells. (**A**) Gelatin degradation assay showing that mouse 4T1 cells form mature invadopodia. Cells were plated on Oregon Green 488-labelled gelatin-coated coverslips for 48 hours, fixed and stained for actin and cortactin to visualize invadopodia (insets). (**B**) Western blot showing CSRP2 protein levels in 4T1 cell lines transduced to stably express a control, non-targeting, shRNA (shCtr) or a CSRP2 transcript-targeting shRNA (shCSRP2). (**C**) Transwell invasion assay. Invading control and CSRP2-depleted 4T1 cells in normoxia and hypoxia at 48 h were quantified via MTT staining. Results were expressed relatively to the invasion of expressing normoxic cells (set to 1). The data originate from 3 independent experiments. (**D**) Actively ECM degrading cells as expressed as percentage of the total cell population. (**E**) Degradation index corresponding to the average of degraded matrix per cell. (**F**) Number of mature invadopodia per cell (as defined by the number F-actin and cortactin co-labelled puncta overlapping with areas of gelatin clearing). The data originate from at least three independent experiments (n ≥ 60 cells). Bars = 15 µm. *$p < 0.05$; **$p < 0.01$, ***$p < 0.001$.

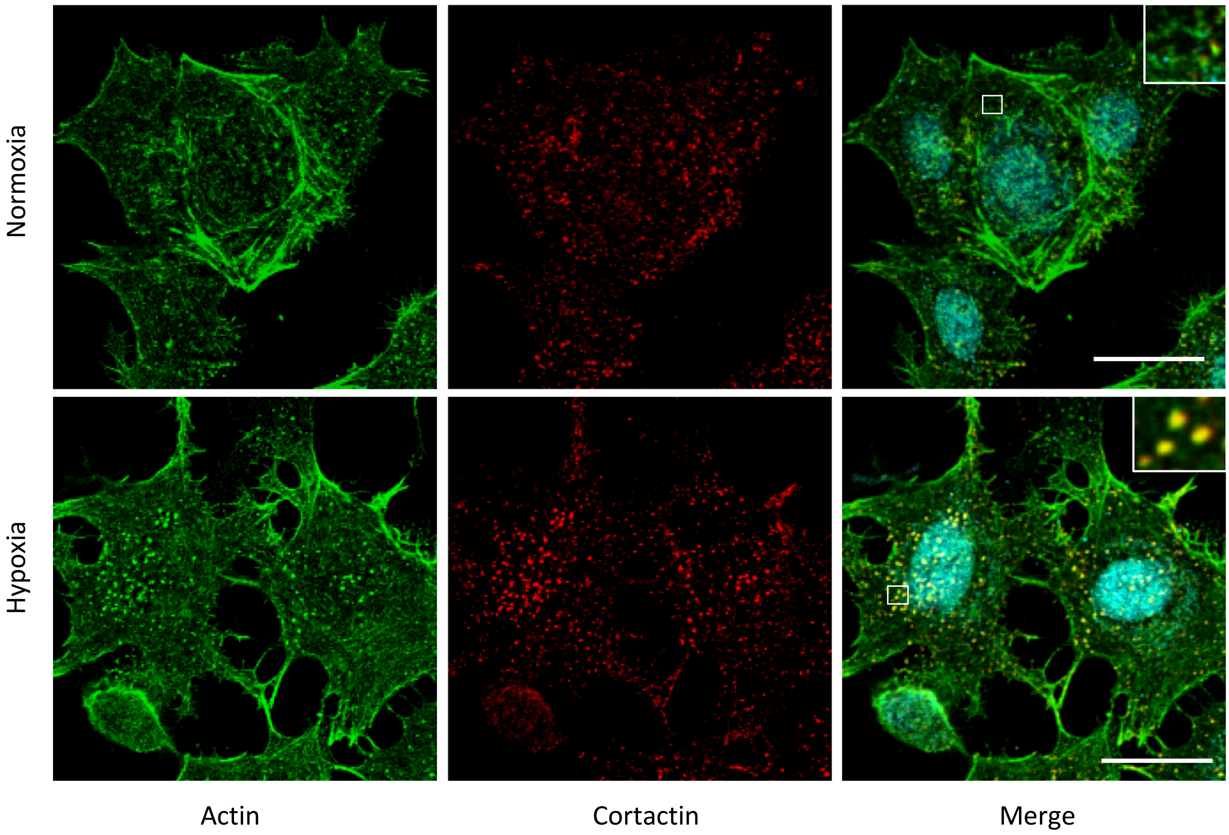

Fig. S4. Hypoxia-induced invadopodium precursors in MCF-7 plated on non-denatured collagen. MCF-7 cells were plated on collagen I-coated slides and incubated for 24 hours in normoxic or hypoxic conditions. After fixation, MCF-7 cells were stained for actin (in green) and cortactin (in red), and imaged using confocal microscopy. The inset shows that only hypoxia induces cortactin relocalization to ventral actin puncta. Bars = 15 µm.

A

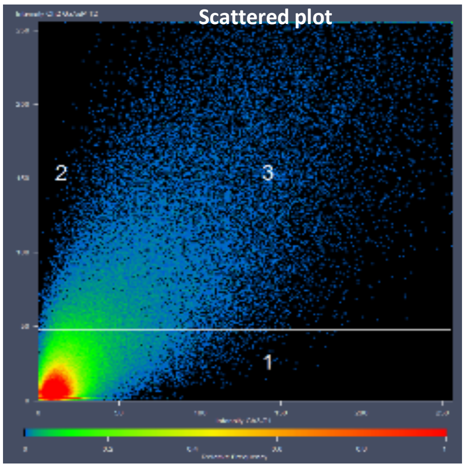

B

MCF7

Mouse 1	Tumor section 1	0.721
	Tumor section 2	0.581
	Tumor section 3	0.726
	Tumor section 4	0.751
	Tumor section 5	0.813
Mouse 2	Tumor section 1	0.737
	Tumor section 2	0.798
	Tumor section 3	0.729
	Tumor section 4	0.74
	Tumor section 5	0.719
Mouse 3	Tumor section 1	0.808
	Tumor section 2	0.792
	Tumor section 3	0.846
	Tumor section 4	0.747
	Tumor section 5	0.769
Mouse 4	Tumor section 1	0.5
	Tumor section 2	0.765
	Tumor section 3	0.738
	Tumor section 4	0.771
	Tumor section 5	0.703
	Tumor section 6	0.584
	Tumor section 7	0.801
	average	0.73+/-0.08
	p-value	6.80E-05

C

MDA-MB-231

Mouse 1	Tumor section 1	0.787
	Tumor section 2	0.717
	Tumor section 3	0.691
	Tumor section 4	0.747
	Tumor section 5	0.726
Mouse 2	Tumor section 1	0.744
	Tumor section 2	0.806
	Tumor section 3	0.691
	Tumor section 4	0.627
	Tumor section 5	0.756
Mouse 3	Tumor section 1	0.709
	Tumor section 2	0.701
	Tumor section 3	0.738
	Tumor section 4	0.723
	Tumor section 5	0.695
Mouse 4	Tumor section 1	0.731
	Tumor section 2	0.824
	Tumor section 3	0.821
	Tumor section 4	0.776
	Tumor section 5	0.802
	Tumor section 6	0.859
average		0.75+/-0.15
p-value		6.67E-05

Fig. S5. CSRP2 and HIF-1α protein levels are highly correlated in MCF-7 and MDA-MB-231 tumour xenografts. (**A**) Example of scattered plots showing pixel intensity for CSRP2 versus HIF-1α signals in an MCF-7 tumour section. (**B and C**) Tables showing the correlation coefficients for CSRP2 and HIF-1α protein levels (extracted from scattered plots) in 22 MCF-7 (B) and 21 MDA-MB-231 (C) tumour xenograft sections. The sections originating from the same tumour were taken from distant sites. An average correlation coefficient value and a corresponding p value are given below each table.

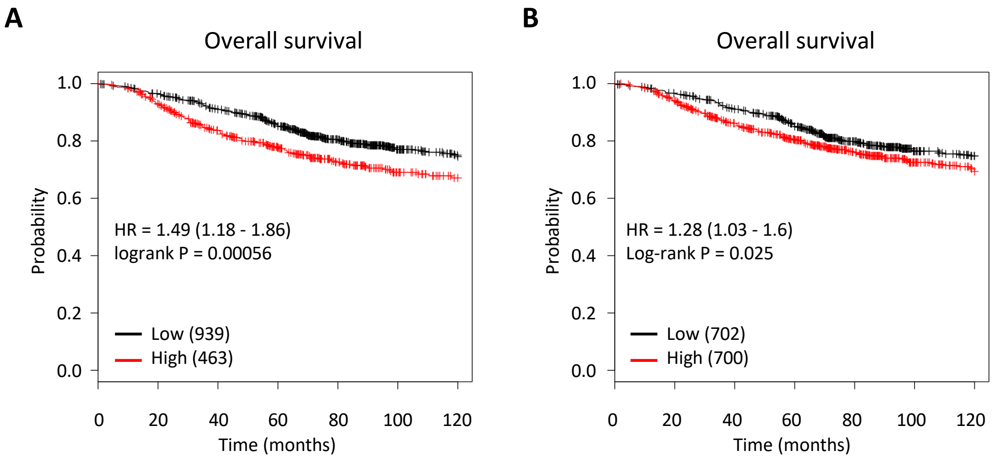

Fig. S6. Kaplan-Meier survival analyses in relation to CSRP2 expression (affy ID 207030s_at) in breast carcinoma using overall survival as an endpoint. Patients were stratified according to the upper (A) or median (B) CSRP2 expression values. The patient samples, hazard ratio with 95% confidence interval, and p value (Logrank test) are displayed on each chart.

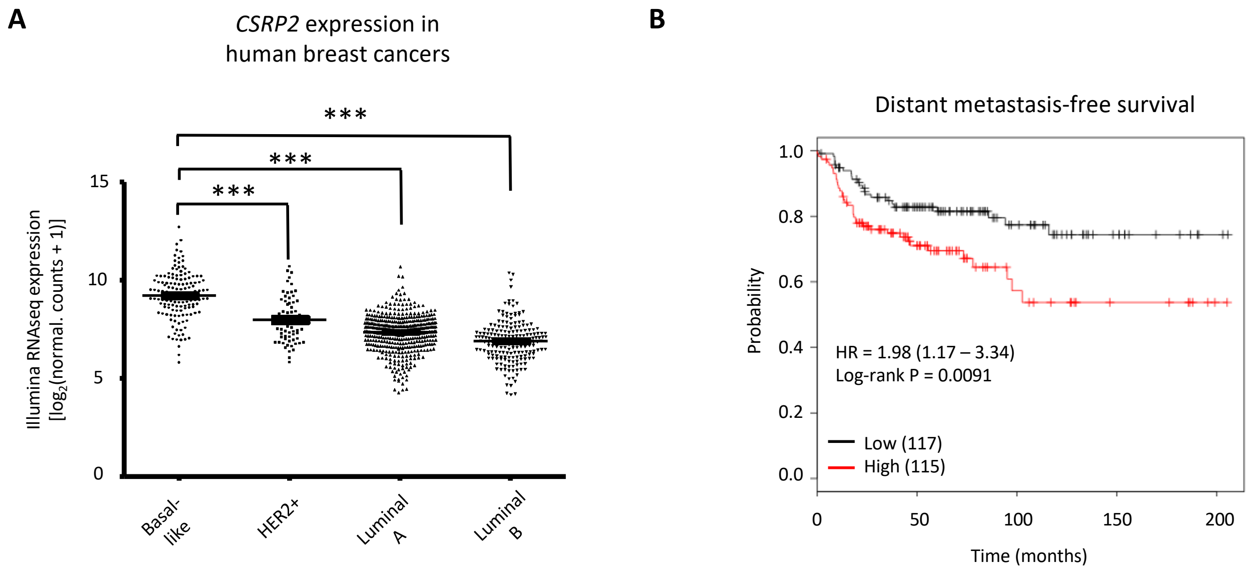

Fig. S7. *CSRP2* levels are elevated and is a stronger predictor of distant metastasis-free survival in basal subtype human breast cancers. (A) *CSRP2* gene expression data for the large TCGA invasive, human breast cancer data set (n = 1,215) was compared across the different breast cancer subtypes and found to be an order of magnitude higher in the basal subtype. (**B**) Kaplan-Meier survival analysis was performed; patients were stratified according to the median *CSRP2* expression value and distant metastasis-free survival was used as an endpoint. The patient samples, hazard ratio with 95% confidence interval, and *p* value (log-rank test) are displayed on the chart. *** denotes a p-value less than 0.001.
